# Empirical determination of breed-of-origin of alleles in three-breed cross pigs

**DOI:** 10.1186/s12711-016-0234-9

**Published:** 2016-08-04

**Authors:** Claudia A. Sevillano, Jeremie Vandenplas, John W. M. Bastiaansen, Mario P. L. Calus

**Affiliations:** 1 Animal Breeding and Genomics Centre, Wageningen University, PO Box 338, 6700 AH Wageningen, The Netherlands; 2 Topigs Norsvin, PO Box 43, 6640 AA Beuningen, The Netherlands; 3 Animal Breeding and Genomics Centre, Wageningen UR Livestock Research, 6700 AH Wageningen, The Netherlands

## Abstract

**Background:**

Although breeding programs for pigs and poultry aim at improving crossbred performance, they mainly use training populations that consist of purebred animals. For some traits, e.g. residual feed intake, the genetic correlation between purebred and crossbred performance is low and thus including crossbred animals in the training population is required. With crossbred animals, the effects of single nucleotide polymorphisms (SNPs) may be breed-specific because linkage disequilibrium patterns between a SNP and a quantitative trait locus (QTL), and allele frequencies and allele substitution effects of a QTL may differ between breeds. To estimate the breed-specific effects of alleles in a crossbred population, the breed-of-origin of alleles in crossbred animals must be known. This study was aimed at investigating the performance of an approach that assigns breed-of-origin of alleles in real data of three-breed cross pigs. Genotypic data were available for 14,187 purebred, 1354 F_1_, and 1723 three-breed cross pigs.

**Results:**

On average, 93.0 % of the alleles of three-breed cross pigs were assigned a breed-of-origin without using pedigree information and 94.6 % with using pedigree information. The assignment percentage could be improved by allowing a percentage (f_r_) of the copies of a haplotype to be observed in a purebred population different from the assigned breed-of-origin. Changing f_r_ from 0 to 20 %, increased assignment of breed-of-origin by 0.6 and 0.7 % when pedigree information was and was not used, respectively, which indicates the benefit of setting f_r_ to 20 %.

**Conclusions:**

Breed-of-origin of alleles of three-breed cross pigs can be derived empirically without the need for pedigree information, with 93.7 % of the alleles assigned a breed-of-origin. Pedigree information is useful to reduce computation time and can slightly increase the percentage of assignments. Knowledge on the breed-of-origin of alleles allows the use of models that implement breed-specific effects of SNP alleles in genomic prediction, with the aim of improving selection of purebred animals for crossbred offspring performance.

**Electronic supplementary material:**

The online version of this article (doi:10.1186/s12711-016-0234-9) contains supplementary material, which is available to authorized users.

## Background

The genetic correlation between purebred and crossbred performance (r_pc_) is a crucial parameter that determines the effect of selection at the nucleus level, where purebred animals are used, on the rate of genetic change at the production level, where crossbred animals are used [1, 2]. In many cases, r_pc_ deviates from 1 because of (1) different genetic backgrounds, and (2) different management procedures for purebreds and crossbreds [1, 3, 4]. As r_pc_ decreases, the benefit of using crossbred information increases [1, 5], e.g. Dekkers [6] reported that even with a r_pc_ as low as 0.7 using crossbred information was advantageous. When information on crossbred animals is used, effects of single nucleotide polymorphisms (SNPs) may be breed-specific because linkage disequilibrium (LD) patterns between a SNP and a quantitative trait locus (QTL) [4] and allele frequencies and allele substitution effects of a QTL may differ between breeds [7]. With genomic prediction, it is possible to determine the effect of alleles from different breeds and, thus, it can be used to select purebred animals for crossbred performance. An additive model that accounts for breed-specific SNP effects for genomic prediction using crossbred information was proposed by Ibánẽz-Escriche et al. [8] and Christensen et al. [9, 10]. Ibánẽz-Escriche et al. [8] and Esfandyari et al. [11] showed with simulated data that, under some conditions (i.e., low SNP density, large training data size, and low breed relatedness), the model that accounts for breed-specific SNP effects outperformed models in which SNP effects are assumed to be the same across breeds. If the above-mentioned conditions that favor the model that accounts for breed-specific effects with simulated data are met in real, then it is important to determine whether such models are also superior in real data.

To estimate the effect of a SNP allele that is present in a crossbred animal and originates from a purebred animal, the breed-of-origin of alleles in crossbreds must be known. While breed-of-origin of alleles was assumed to be known without error by Ibánẽz-Escriche et al. [8] and Esfandyari et al. [11], errors in breed-of-origin of alleles and the total percentage of alleles assigned to a breed-of-origin likely impact the accuracy of subsequent analyses such as genomic prediction.

For a two-way cross, determining the breed-of-origin of alleles is relatively easy, especially when both parents are genotyped [12]. However, in pig and chicken production, three-way crosses are commonly used. Bastiaansen et al. [4] developed an approach to assign breed-of-origin to alleles in three-breed cross animals. They used a long-range phasing method [13] to relax the dependency on genotyped parents and available pedigree information. Haplotypes that were derived from the long-range phasing method were assigned to a breed if they were present in only one of the purebred populations, which subsequently allowed assigning the breed-of-origin of alleles when that haplotype was observed in crossbred animals. Vandenplas et al. [14] improved and tested the approach to assign breed-of-origin of alleles on simulated data and obtained highly accurate allele assignments in three-breed cross animals without using pedigree information. Our aim was to investigate the performance of assignment of breed-of-origin of alleles on real data of three-breed cross pigs. The impact of using pedigree information on the crossbred animals on the assignment of breed-of-origin of alleles was also tested because in this dataset the pedigree was completely known and this approach is able to use such information when available.

## Methods

### Ethics statement

The data used for this study was collected as part of routine data recording in a commercial breeding program. Samples collected for DNA extraction were only used for routine diagnostic purposes of the breeding program. Data recording and sample collection were conducted strictly in line with the Dutch law on the protection of animals (Gezondheids- en welzijnswet voor dieren).

### Genotypic data

**Fig. 1:**
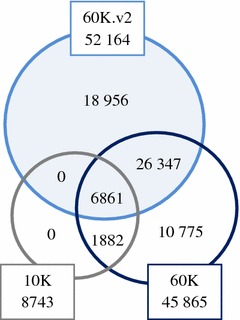
Distribution of SNPs across the three different SNP panels after pruning. SNPs within the *shadowed blue circle* are included in the final set of SNPs. SNPs outside the *shadowed blue circle* were used during the imputation procedure

We used pigs that originated from a three-way crossbreeding design, in which Landrace (LR) pigs were crossed with Large White (LW) pigs to produce F_1_ (LR × LW or LW × LR) crossbred pigs, which in turn were crossed with synthetic boar (S) pigs to produce three-breed cross pigs [S (LR × LW) or S (LW × LR)]. Genotyping data was available for 14,187 purebred, 1354 F_1_, and 1723 three-breed cross pigs (Table 1). All pigs were genotyped using one of the three following SNP panels: Illumina PorcineSNP60.v2 BeadChip (60K.v2), Illumina PorcineSNP60 BeadChip (60K), or Illumina PorcineSNP10 BeadChip (10K) (see Table 1 for details). LR, LW and S pigs were primarily genotyped with the 60K (N = 2352), 10K (N = 3618), and 10K (N = 1233) chips, respectively. F_1_ pigs were primarily genotyped with the 60K.v2 (N = 786) chip and three-breed cross pigs with the 10K (N = 1432) chip. SNPs were removed from the data if they had the same position as another SNP (only one removed), if they had no position assigned, or if they were present on *Sus scrofa* chromosome (SSC) X or SSCY. The SNP set for subsequent analyses consisted of SNPs from the 60K.v2 that had a call rate higher or equal to 90 % across all purebred lines. Pigs genotyped with the 60K or 10K chips were imputed to the 60K.v2 panel. SNPs with low imputation accuracy across all purebred lines and F_1_ crossbreds (concordance <0.80) were removed from the final set of SNPs. Finally, 52,164 SNPs remained for the analyses (Fig. 1).

**Table 1:** Number of genotyped pigs available per SNP panel, and per purebred line or cross

SNP panel	Synthetic boar (S)	Landrace (LR)	Large White (LW)	F1 (LR × LW) (LW × LR)	3-breed cross [S (LR × LW)] [S (LW × LR)]	Total
60K.v2	810	914	878	786	0	3388
60K	782	2352	2687	543	291	6655
10K	1233	913	3618	25	1432	7221
Total	2825	4179	7183	1354	1723	17,264

### Imputation

FImpute Version 2.2 software [15] was chosen for imputation with default parameter settings and using pedigree information because it is one of the most efficient available software programs for imputation [15, 16]. Within each of the three purebred lines, LR, LW, and S, imputation was performed in two steps: (1) pigs genotyped with the 10K chip were imputed to 60K, and (2) all pigs with 60K data were imputed to 60K.v2. For F_1_ and three-breed cross pigs, imputation was done in a single step, i.e. pigs genotyped with the 10K and 60K chips were directly imputed to 60K.v2, because all ancestors were genotyped or already imputed to 60K.v2. The numbers of SNPs from each panel that were used in each imputation step are in Fig. 1.

### Validation of imputation

Imputation accuracy was assessed in 80 pigs from each of the purebred lines, LR, LW, and S, and in 80 F_1_ crossbred pigs, which were all genotyped with the 60K.v2 panel. Accuracy of imputation in three-breed cross pigs was not assessed because none of them were genotyped with the 60K.v2. All pigs that were chosen to assess imputation accuracy had no offspring and both their parents were genotyped with the 60K.v2, 60K, or 10K chips. In these pigs, the genotypes of all SNPs on the 60K.v2 panel were set to missing, except for the SNPs that were also on the 10K panel. Imputation accuracy was calculated for each SNP in two ways, based on concordance and Pearson correlation, using the real and imputed genotypes. Pearson correlations per SNP between the real and imputed genotypes were corrected for minor allele frequency (MAF), i.e., ${\text{real genotype}}-2{\text{*MAF}}$ and ${\text{imputed genotype}}-2{\text{*MAF}}$. The MAF for each SNP was calculated using the data for the 80 pigs tested from each population. SNPs with low imputation accuracy across all purebred lines and F_1_ crossbreds (concordance <0.80) were removed from the final set of SNPs.

### Assignment of breed-of-origin of alleles

To assign breed-of-origin of alleles to three-breed cross pigs, we used an approach that consisted of three steps: (1) phasing the haplotypes of both purebred and crossbred pigs, (2) determining the unique haplotypes among the pure breeds, and (3) assigning the breed-of-origin for each allele carried on the haplotypes of crossbred pigs, i.e. F_1_ and three-breed cross pigs. For these steps, we used all the 52,164 SNPs in the final set.

#### Phasing

AlphaPhase1.1 software [13] that implements a long-range phasing and haplotype library imputation algorithm was used to phase the genotypes. Although FImpute [15] also searches for long shared haplotypes and builds a haplotype library, the breed-of-origin approach cannot use this program because it also searches for short shared haplotypes. Short shared haplotypes can be difficult to assign to a breed because they may be shared across breeds. Long-range phasing is also of particular interest because it does not rely on pedigree information. However, we tested both scenarios, phasing with and without pedigree information, to assess if allele assignment was improved when using pedigree information. Due to computational limitations, assigning breed-of-origin without using pedigree information was performed only for chromosomes 3, 4, 9, 12, and 16. For both scenarios, haplotypes were built using nine combinations of core and tail lengths: 350:50, 250:100, 300:100, 350:100, 150:200, 200:200, 250:200, 300:200, 350:200. The concepts of core and tails are outlined in detail in Hickey et al. [13]. Briefly, a core is a consecutive string of SNPs that are phased simultaneously, while tails are consecutive strings of SNPs that are immediately adjacent to either end of a core and that are used together with the core SNPs to identify which pigs in the data carry the same haplotype. Each combination of core and tails was run both considering “Offset” and “NotOffset” modes. The “Offset” mode shifts the start of the cores to halfway along the first core, creating 50 % overlaps between cores. These settings were chosen based on results of Vandenplas et al. [14] and allowed each allele to be considered 18 times through different haplotypes of variable length. Varying the haplotype lengths may improve the overall phasing when some animals do and others do not have close relatives present in the genotype data. For all phasing analyses, 1 % of genotype errors and 1 % disagreement between genotypes and haplotypes were allowed.

#### Assignment of breed for haplotypes and alleles

Assignment of breed-of-origin to haplotypes was performed as in Vandenplas et al. [14]. To assign a breed-of-origin to a haplotype, it was necessary that most of its copies were present in a specific breed. We tested two relaxation factors (f_r_), i.e. 0 and 20 %, which is the maximum percentage of the copies of a haplotype that may be observed in a different purebred population. When the percentage of copies of a haplotype that was observed in a single breed was less than (100 − f_r_) %, the breed-of-origin for that haplotype was set to unknown.

Assignment of breed-of-origin to each allele that is carried on the haplotypes of crossbred animals is based on the knowledge available for the breed-of-origin of the haplotypes, the zygosity (i.e., homozygosity or heterozygosity) of the locus, and the breed composition of the crossbred animals (see Vandenplas et al. [14] for the algorithm). Each allele at each locus can receive 18 breed-of-origin assignments, but, in some analyses, this number can be smaller when no breed is assigned to the haplotype.

### Principal component analysis

A principal component analysis (PCA) was performed to check if three-breed cross pigs with a low assignment of breed-of-origin to their alleles were genetically distinct to the three-breed cross population. The PCA was performed by eigen decomposition of the genomic relationship matrix (**G-**matrix). The **G**-matrix was computed as in Yang et al. [17], using our in-house software calc_grm [18].

## Results

### Imputation and accuracies of imputation

**Fig. 2:**
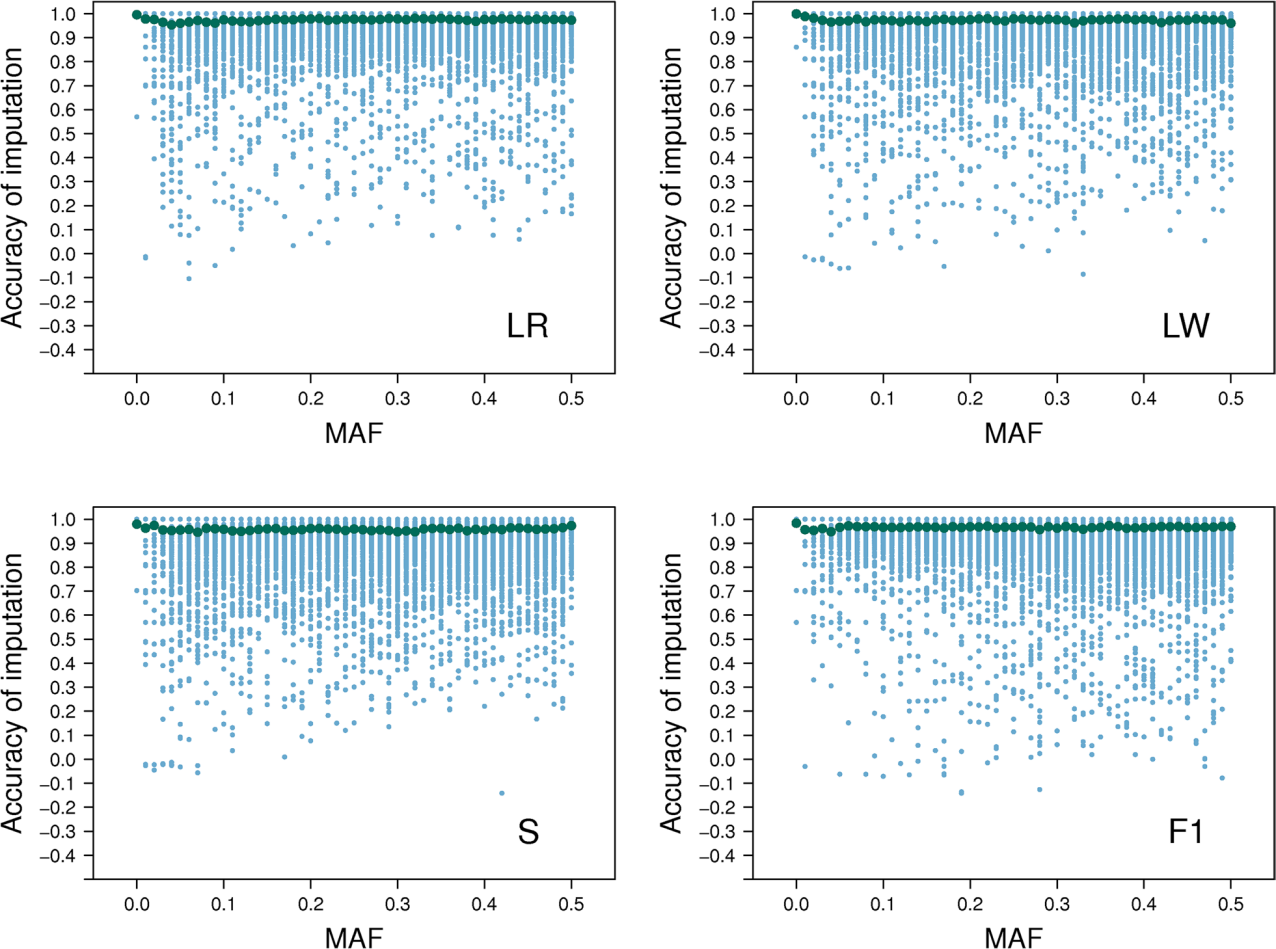
Accuracy of imputation according to minor allele frequencies. Minor allele frequencies (MAF) of genotyped SNPs versus the accuracy (Pearson correlation) of imputation from the PorcineSNP10 BeadChip panel to Illumina PorcineSNP60.v2 BeadChip for 80 pigs of each purebred line, i.e. synthetic boar (S), Landrace (LR), Large White (LW), and crossbred F_1_ pigs. The *dark green dots* are the average accuracy for different MAF

Accuracies of imputation were very close to 1, both when based on concordance and Pearson correlation (Table 2). The Pearson correlation between imputed and real genotypes per SNP was greater than 0.96 across all pure lines and F_1_ pigs (Table 2). The Pearson correlation per SNP was very similar across different MAF (Fig. 2). Some individual SNPs (N = 406) showed poor imputation accuracy (concordance <0.80) and were removed from the set of SNPs. The final set of SNPs considered for imputation and assignment of breed-of-origin for alleles of three-breed cross pigs included 52,164 SNPs from the 60K.v2 panel.

**Table 2:** Average imputation accuracies computed across pigs or SNPs

	Pig	SNP	
	Concordance	Correlation	Concordance
Landrace	0.99	0.97	0.98
Large White	0.99	0.97	0.98
Synthetic boar	0.98	0.96	0.98
F1 crossbred	0.98	0.97	0.98

Accuracy was computed for the masked loci as the proportion of pigs or loci that had the same observed and imputed genotype (concordance), or the same Pearson correlation between the observed and imputed genotypes

### Assignment of breed-of-origin for alleles

#### Comparison of different settings used for assignment of breed-of-origin

All pigs were used to assign the breed-of-origin of alleles but the results are presented only for three-breed cross pigs. Breed-of-origin assignments were obtained from analyses without pedigree information for chromosomes 3, 4, 9, 12, and 16, and from analyses with pedigree information for all autosomes. For chromosomes 3, 4, 9, 12, and 16, on average 93.0 % (±1.0 %) of the alleles of a three-breed cross pig were assigned to a breed-of-origin without using pedigree information and 94.6 % (±1.0 %) with using pedigree information, both with a relaxation factor (f_r_) equal to 0 % (Table 3). For all autosomes, on average 93.9 % (±1.4 %) of the alleles of a three-breed cross pig were assigned to a breed-of-origin when using pedigree and f_r_ set at 0 %. Relaxing f_r_ from 0 to 20 % increased the assignment by 0.6 and 0.7 % with and without using pedigree information, respectively, for chromosomes 3, 4, 9, 12, and 16, and increased the assignment by 1.3 % with using pedigree information for all autosomes (Table 3). In general, increases in assignment percentage were small regardless of whether pedigree information was used or not or whether f_r_ was set to 0 or 20 %.

**Table 3:** Allele assignment (%) to purebred lines as breed-of-origin in four scenarios

Pedigree	f_r_ (%)	Paternal	Maternal			Total
		Line S	Line LR	Line LW	Total	
No^a^	0	49.5 (0.25)	22.4 (0.59)	21.1 (0.38)	43.5 (0.80)	93.0 (1.04)
	20	49.6 (0.23)	22.5 (0.64)	21.6 (0.42)	44.1 (0.82)	93.7 (1.03)
Yes^a^	0	49.7 (0.26)	23.2 (0.48)	21.8 (0.33)	45.0 (0.71)	94.6 (0.97)
	20	49.7 (0.25)	23.0 (0.61)	22.6 (0.83)	45.5 (0.67)	95.2 (0.91)
Yes^b^	0	49.5 (0.46)	22.5 (0.90)	21.8 (0.53)	44.4 (1.13)	93.9 (1.44)
	20	49.6 (0.42)	23.0 (0.65)	22.7 (0.59)	45.7 (0.73)	95.2 (0.95)

Allele assignment to synthetic boar (S), Landrace (LR), or Large White (LW) for four scenarios, when pedigree information is used or not, and with a relaxation factor (fr) of 0 or 20 %

SD are in parenthesis

^a^Averages estimated using chromosomes 3, 4, 9, 12, and 16

^b^Averages estimated using all 18 autosomes

The assigned breed-of-origin of alleles for heterozygous genotypes may differ depending on the approach used. To assess the effect of using pedigree information, breed-of-origin assignments with or without the use of pedigree information were compared. Both scenarios included only chromosomes 3, 4, 9, 12, and 16 (Table 4, comparison A). Only 0.3 % of the assignments displayed a change in their breed-of-origin depending on the use of pedigree information or not. Assignments were concordant for 94.2 % of the genotypes and 5.5 % of the genotypes were assigned a breed-of-origin by only one of the two approaches.

**Table 4:** Comparison between different scenarios for the assignment of breed-of-origin of alleles

Comparison A			Comparison B		
Pedigree	No pedigree	%	f_r_ 20 %	f_r_ 0 %	%
Concordance		94.16	Concordance		99.24
Assigned	Not assigned	3.57	Assigned	Not assigned	0.63
Not assigned	Assigned	1.97	Not assigned	Assigned	0.07
Disagreement		0.30	Disagreement		0.06

(A) Breed-of-origin approach with versus without pedigree (relaxation factor (f_r_) of 0 %)

(B) Breed-of-origin approach with f_r_ set to 20 % versus f_r_ set to 0 % (with pedigree)

Concordance, same allele assigned to the same breed-of-origin by both scenarios or same allele not assigned to a breed-of-origin by both scenarios

Disagreement, same allele assigned to different breed-of-origins by both scenarios

Allele assigned to a breed-of-origin by only one scenario (assigned–not assigned or not assigned–assigned)

To assess the impact of increasing the relaxation factor, assignments of breed-of-origin obtained with f_r_ set at 0 and 20 % were compared. In this case, both scenarios included pedigree information and only chromosomes 3, 4, 9, 12, and 16 were used (Table 4, comparison B). Only 0.1 % of the assignments displayed a change in their breed-of-origin between setting f_r_ at 0 or 20 %. The assignments were concordant for 99.2 % of the genotypes and 0.7 % of the genotypes were assigned a breed-of-origin by only one of the approaches. Because differences in breed-of-origin assignments between options were small, only results obtained with pedigree information and an f_r_ set at 20 % will be presented in the following.

#### Performance of assignment of breed-of-origin

**Fig. 3:**
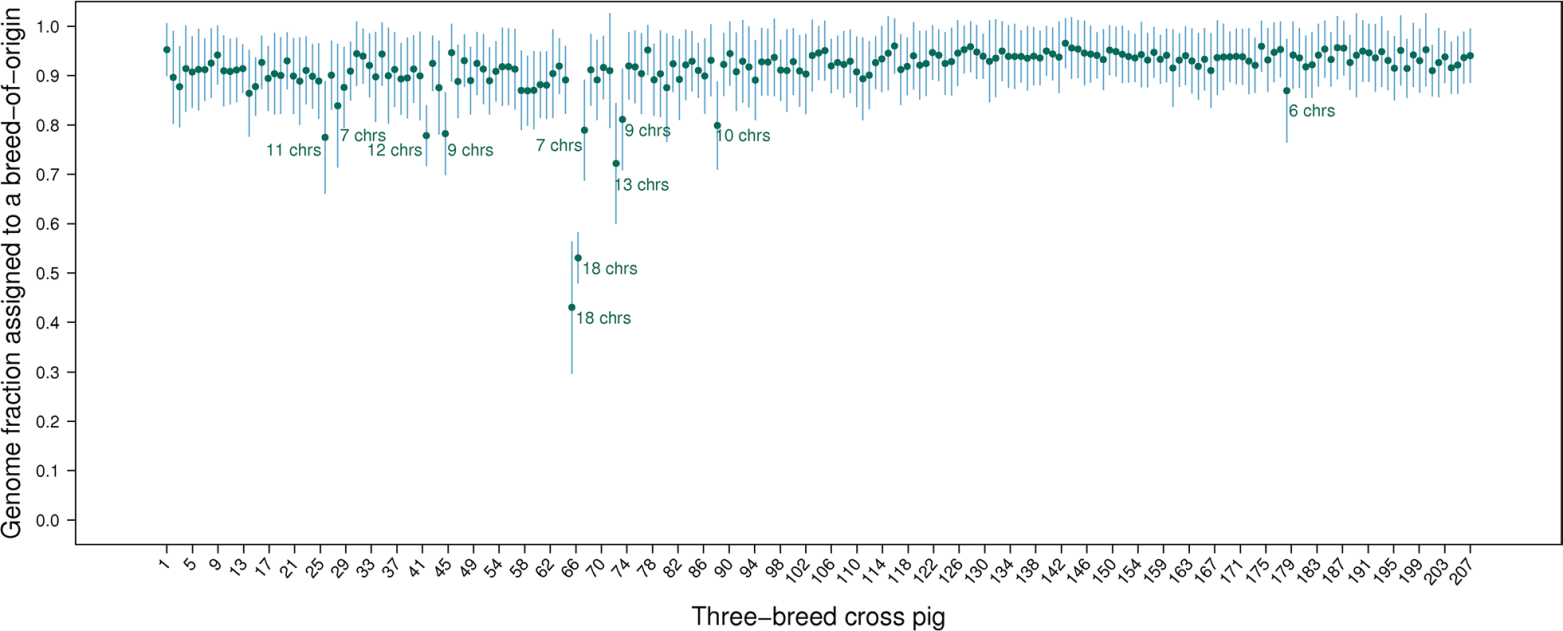
Average (±SD) assignment of breed-of-origin of alleles for 207 three-breed cross pigs. All three-breed cross pigs had at least one of their chromosomes with less than 80 % breed-of-origin assignment of alleles. *Numbers* of chromosomes per pig with poor assignment are written next to the averages (number is omitted if number of chromosomes is smaller than 5)

Average assignment percentages were similar across three-breed cross pigs. On average, for each chromosome, at least 80 % of alleles were assigned a breed-of-origin to 98.7 % of the three-breed cross pigs. Of the three-breed cross pigs, 8 % (N = 141) had a chromosome for which less than 80 % of the alleles were assigned and 4 % (N = 66) had multiple such chromosomes. The assignment percentage of these 207 three-breed cross pigs is illustrated in Fig. 3. The chromosome that has the lowest percentage of assignment varied across these 207 pigs. The lowest assignment for a chromosome was observed in a three-breed cross pig for which only 19.0 % of the alleles on chromosome 9 were assigned to a breed. For this pig, chromosome 6 had the highest assignment, for which 67 % of the alleles were assigned to a breed. Two three-breed cross pigs, including the one mentioned above, had a low percentage of assignment for all 18 chromosomes (Fig. 3).

The average assignment of breed-of-origin of alleles was similar across chromosomes, with a standard deviation (SD) of 0.95 % among the 18 chromosomes. Within chromosome, the SD ranged from 3.36 % for chromosome 1 and 13, to 5.97 % for chromosome 2. The highest assignment was obtained for chromosome 17 (96.5 %) and the lowest for chromosome 12 (93.6 %) (see Additional file 1). For chromosome 17, 49.8 % of the alleles were assigned to the paternal S purebred line, 23.1 % to the maternal LR purebred line, and 23.6 % to the maternal LW purebred line. For chromosome 12, 49.3 % of the alleles were assigned to the paternal S purebred line, 21.7 % to the maternal purebred LR line, and 22.6 % to the maternal LW purebred line. The main differences between chromosomes were due to differences in the percentage assigned to the maternal purebred lines.

**Fig. 4:**
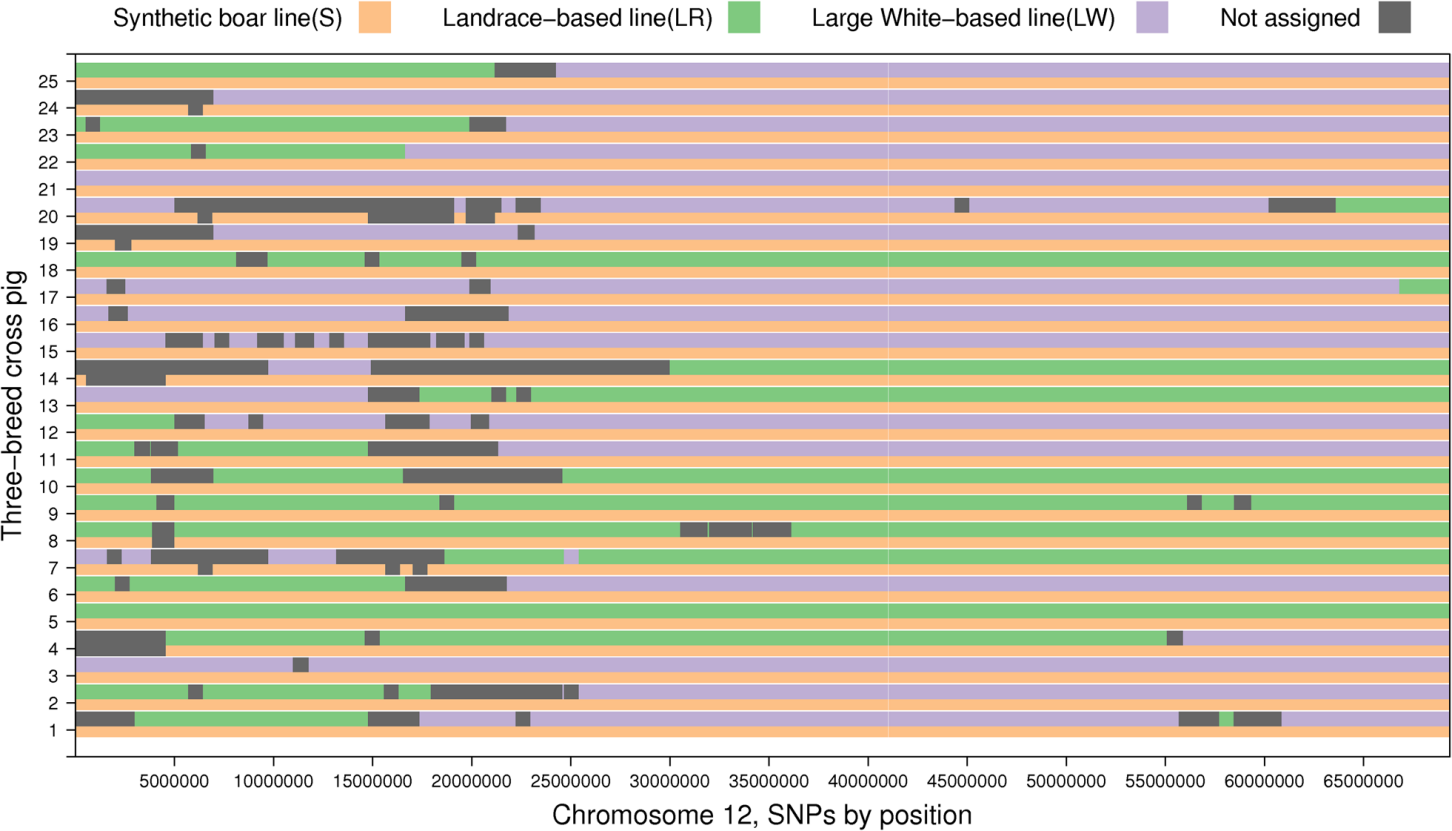
Breed-of-origin of alleles in 25 three-breed cross pigs. Each three-breed cross pig is represented in two rows, one row representing the paternal and one row the maternal chromosome. *Dark grey regions* indicate unassigned allelic origin. *White regions* indicate regions that not covered with SNPs

For most three-breed cross pigs, one chromosome of each pair was almost completely assigned to the paternal S purebred line, as shown for 25 random pigs in Fig. 4, while the other chromosome showed large blocks that were assigned to the maternal LR or LW purebred line. While it is expected that 50 % of the maternal chromosome originates from one of the two maternal purebred lines, these percentages can deviate strongly from this value for individual animals. The pattern in Fig. 4 is as expected based on the 1.2 recombination rate of chromosome 12 [19], and we observed on average one recombination per chromosome. However, near the ends of the maternal chromosomes, the number of alternate assignments of breed-of-origin of alleles between the maternal LR or LW purebred lines increased, which is consistent with the higher levels of recombination that are observed in these chromosome regions [19]. Assignment of breed-of-origin to each allele is also based on the breed composition of the crossbred animals. For one three-breed cross pig, if the origin of the maternal allele is assigned, the algorithm always assigns the paternal origin to the other allele at the same locus, i.e. in Fig. 4 no dark grey region is observed opposite to an assigned maternal allele. The other way around, if the origin of the paternal allele is assigned, the algorithm does not necessarily always assign the maternal origin to the other allele at the same locus, because it cannot choose between the two maternal purebred lines, as can be observed from dark grey regions opposite to an assigned paternal allele.

#### Principal component analysis

**Fig. 5:**
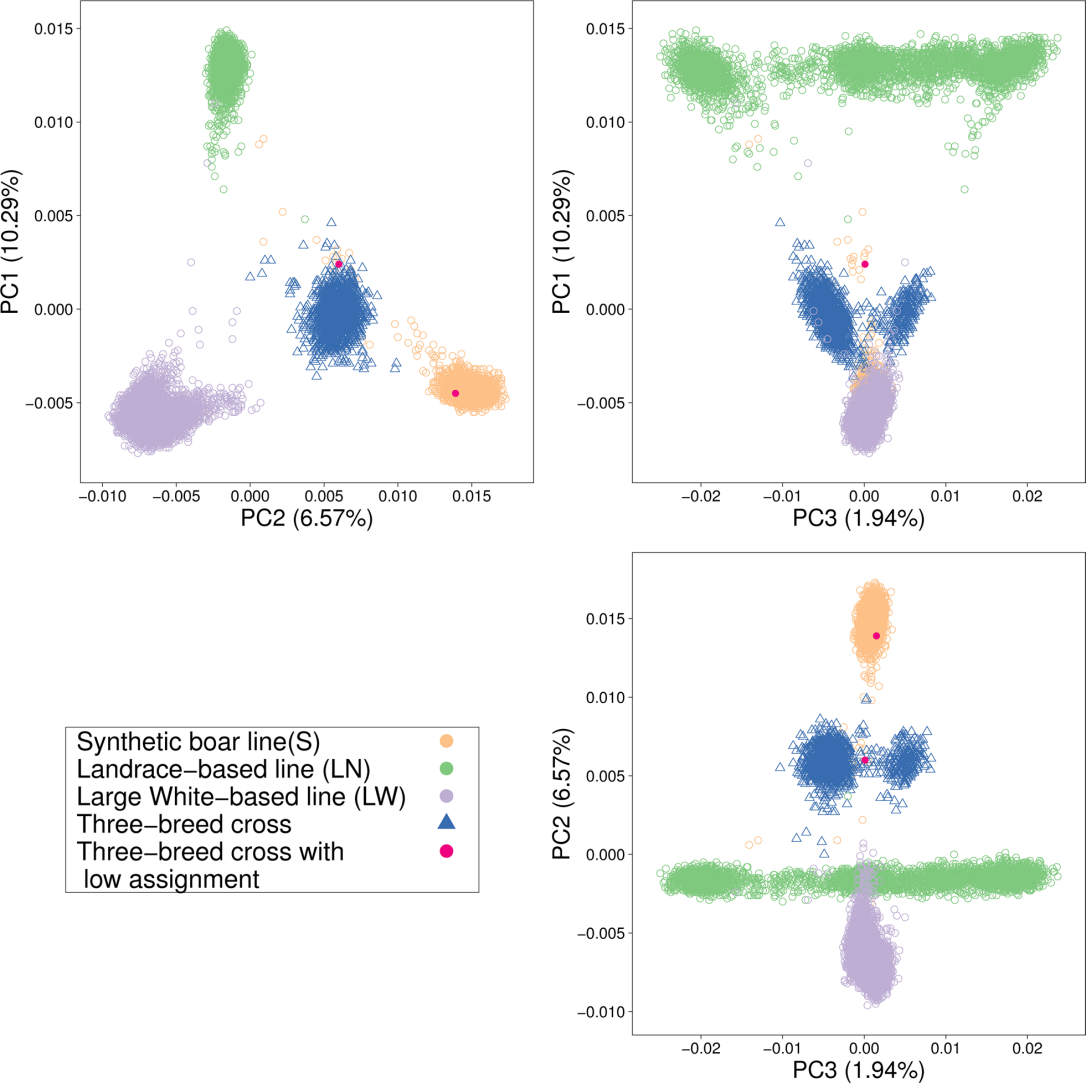
Three first principal components (PC) for the three purebred lines and three-breed cross pigs. Each *circle* (o) or *triangle* (∆) represents a pig. The *two pink dots* represent the two three-breed cross pigs with a low percentage of assignment for all 18 chromosomes

The principal component analysis of the genomic relationship matrix provided a clear separation between the purebred lines and between the three-breed cross pigs (Fig. 5). The first and second principal components together explained 16.9 % of variation, while the third principal component only explained 1.9 % of the variation, which is mainly associated with variation within the LR purebred line population. Previously, we detected two three-breed cross pigs with a low percentage of assignment for all 18 chromosomes. In Fig. 5, we plotted the first three principal components of the genomic relationship matrix and we observed that one of these pigs was placed within the paternal S purebred line population, while the other pig was placed outside the three-breed cross population, but also outside all purebred line populations. This indicates that these two pigs were genetically distinct from the three-breed cross population.

## Discussion

### Imputation

For the three purebred lines, LR, LW, and S, imputation was performed in two steps, 10K genotypes were imputed to 60K, and the output of the first step was imputed to 60K.v2. This strategy was chosen because the 10K panel shared more SNPs (8743) with the 60K panel than with the 60K.v2 panel (6861). Pedigree information was used for the imputation because it was available. However, in the absence of pedigree information and with high-density panels, family information can be captured by searching for long haplotypes and used for imputation [15]. The accuracies of imputation that were obtained in our study, using related pigs that were genotyped with high-density panels (60K or 60K.v2) and using pedigree information, were close to accuracies reported in the literature with similar datasets [16, 20]. Gualdron Duarte et al. [20], imputed 9K genotypes of F_2_ individuals from a Duroc × Pietrain population to 60K, and obtained an accuracy of imputation higher than 0.94. With our data, the accuracy of imputation per SNP was very similar across different values of MAF, which indicates that rare variants were also accurately imputed. Similarly, Gualdron Duarte et al. [20] observed that SNPs with a MAF lower than 0.10 were imputed with reasonably good accuracy in the F_2_ population. Ventura et al. [16], imputed crossbred beef cattle from 6K to 50K, and concluded that the accuracy of imputation of crossbred animals can be high if the number of reference animals genotyped with high-density panels is sufficiently large and if all breeds that have led to the crossbred animals are included. They also used the FImpute software [15] and obtained imputation accuracies higher than 0.94. However, accuracy of imputation was based only on concordance. Concordance estimates for imputation accuracy are generally higher than Pearson correlations. Imputation errors are generally due to the assignment of the major instead of the minor allele, and the probability of such errors decreases as MAF decreases. Therefore, SNPs with a low MAF generally show high concordance [21]. Moreover, the slightly lower accuracies reported by Ventura et al. [16] compared to those found in our study, can be explained by the fact that they lacked pedigree information. Another reason may be the higher levels of genomic divergence between the reference population and the group of animals to be imputed. In addition, the structure of the populations may have also contributed to this difference since pig breeding populations have a small effective population size, few boars with large family sizes, and generally complete pedigree information, while beef cattle populations have a larger number of sires with smaller family sizes and incomplete pedigree information [16]. Accuracy of imputation in our three-breed cross pigs data was not assessed because none of these animals were genotyped with the 60K.v2 chip. However, we would expect high imputation accuracies, i.e. similar to the results obtained for the purebreds and F_1_ pigs. Shared haplotypes should have been found easily and accurately because the reference group was large and related to the target group [15]. Moreover, high imputation accuracy of rare variants was also expected in the three-breed cross pigs, because alleles present in the crossbreds must be present in the purebred parental lines [20].

### Assignment of breed-of-origin

Percentage of assignment of breed-of-origin to alleles increased only slightly when pedigree information was used (1.6 % with f_r_ set at 0 %, and 1.5 % with f_r_ set at 20 %). Using pedigree information is recommended, first to increase allelic assignments, and second to reduce computation time during the phasing analyses. Only a small difference in assignment of breed-of-origin between using pedigree information or not was expected, because this information was only used for the phasing step, and it has been shown that long-range phasing, as implemented in AlphaPhase1.1 software, performs well in the absence of pedigree information [13]. Percentages of assignments were in line with the results based on simulated data that were reported by Vandenplas et al. [14]. In their simulation study [14], the distantly-related breeds scenario is comparable to our real data analysis. We obtained the highest percentage of assignment when using pedigree information and f_r_ equal to 20 %. Based on the simulation study of Vandenplas et al. [14], relaxing the maximum percentage of copies of the haplotype observed in another purebred population from 0 to 10 %, and then to 20 %, slightly increased the percentage of correct assignments but did not influence the percentage of incorrect assignments, and consequently slightly decreased the percentage of unknown assignments for crossbred animals that originated from distantly-related breeds. Across our results in Tables 3 and 4, 91 % of the alleles were always assigned and 2.8 % were never assigned, regardless of whether pedigree information was used or not. Therefore, 6.2 % of the alleles might switch from not being assigned to being assigned or vice versa, depending on whether pedigree information is used or not and the value set for f_r_. Furthermore, we observed that assignments of breed-of-origin obtained with f_r_ set at 0 or 20 % were consistent. Therefore, relaxing the maximum percentage of copies of the haplotype to be observed in another purebred population from 0 to 20 % did appear to have resulted in extra assignments rather than rearrangement of assignments.

### Animals with a low percentage of assignment of breed-of-origin

The percentage of assignment of breed-of-origin to alleles was high and constant across chromosomes. Two hundred and seven three-breed cross pigs had at least one chromosome for which less than 80 % of the alleles were assigned. It is difficult to characterize these animals, since 115 of these have only one or none of their parents genotyped. However across the whole data, 221 three-way crossbred animals also had only one or none of their parents genotyped, which means that 106 of them still achieved more than 80 % assignment for all chromosomes. Relatedness within these three-way crossbred animals does not seem to be the issue either. We found, a maximum of 16 half- or full-sibs (in the scenario with common sire A) and 11 half- or full-sibs (in the scenario with common sire B), however, sires A and B also produced 13 and 15 other half- or full-sibs, respectively, with more than 80 % assignment for all chromosomes. A low assignment percentage was found for the whole genome for two three-breed cross pigs. A principle component analysis of the genotype data showed that these two pigs do not overlap with the three-breed cross population. Thus, the approach used was not able to assign an origin to most of their haplotypes. We suspect that this absence of overlap of these two three-breed cross pigs with the three-breed cross population may be due to erroneous pig identification, i.e. the first pig might have originated from the paternal S purebred line and the second pig from a cross with another line that was not included in this study. This absence of overlap with the corresponding population was also observed for some purebred line pigs, likely for the same reasons. Pigs with low assignment of breed-of-origin to alleles along the whole genome should be removed from the dataset because they do not add information about breed-of-origin of alleles when it is used in further analyses, and because the low assignment may indicate an error in the data. The assignment of breed-of-origin to alleles of other three-breed cross pigs in the dataset should not be affected by these apparently incorrectly labelled pigs, even if the incorrect assignment occurs for the purebred line pigs, i.e. using breed-of-origin assignment with a f_r_ of 20 %, we still expected that at least 80 % of the alleles from the other purebred line pigs would be assigned the correct breed.

With the third principal component, we observed that pigs from the LR line were more variable compared to those from the other purebred lines (Fig. 5). This is probably because the recent history of the LR pigs used in our study involves animals that originated from two populations. As a result, the three-breed cross pigs were also sub-divided into two sub-groups, which probably depended on which of these two sub-populations the LR grand-dam came from. This variation within the LR population was mainly captured by the third principal component but it explained only 1.94 % of the extra variation.

### Phasing and haplotype library

The first step to assign the breed-of-origin of alleles, was to phase the genotypes using the long-range phasing and haplotype library algorithm AlphaPhase1.1 (13). Phasing using pedigree information was on average three times faster than phasing without pedigree information. For the starting analysis, which includes phasing of the purebred animals, and the first batch of the crossbred animals, the assignment of breed-of-origin can still be accurately obtained without pedigree information, but one has to account for the increased computational demand. AlphaPhase1.1 builds a library of all unique haplotypes that long-range phasing has found in the dataset. This library can then be used in subsequent analyses for phasing new crossbred animals that are added to the dataset and that may or may not have pedigree information, without the need to phase the reference population again. Hickey et al. [22] tested this phasing strategy with simulations and obtained 81 to 94 % of correctly phased SNPs with a low error rate (<0.08 %). This phasing strategy can be applied for breed-of-origin assignment to speed up the assignment of alleles of new crossbred animals that are added to the dataset.

### Application

Using crossbred performance for genetic predictions could be beneficial in breeding systems where production animals are crossbred, especially for traits with a low genetic correlation between purebred and crossbred performance. Genomic selection outperforms selection based on pedigree relationships and allows the use of crossbred performance information, even when pedigree information is not available. However, when using crossbred information for genomic prediction, we must take into account that effects of SNPs may be breed-specific because LD patterns between a SNP and a QTL may differ between breeds [4], and allele frequencies and allele substitution effects of QTL may also differ between breeds [7]. To include these differences between breeds in a prediction model, we first need to determine the breed-of-origin of alleles in three-breed cross animals with high accuracy, as in this study, and then use prediction models that estimate breed-specific SNP effects, as proposed by Ibánẽz-Escriche et al. [8] and Christensen et al. [9]. The benefit of this approach, training with crossbred data and using breed-specific SNP effects models, is that allele substitution effects of purebred alleles will be estimated against the genetic background that they will be expressed in. Thus, this approach can potentially incorporate the additive components of dominance and epistasis [8, 23]. This could be used in combination with reciprocal recurrent selection [1] using phenotypes and genotypes of crossbred animals instead of only phenotypes [23]. Under some conditions (i.e., low SNP density, large crossbred training data size, and low breed relatedness), Ibánẽz-Escriche et al. [8] and Esfandyari et al. [11], reported improved predictions using a model that accounts for the breed-of-origin of alleles compared to an additive or dominance model where SNP effects are assumed the same across breeds. In Ibánẽz-Escriche et al. [8] and Esfandyari et al. [11], simulated data were used and breed-of-origin of alleles was assumed to be known a priori. With the results obtained in our study, the genomic model that accounts for breed-of-origin of alleles can be tested with real data. Since applications of genomic prediction require frequent re-estimation of SNP effects to maintain prediction accuracy, genomic prediction based on crossbred performance and breed-of-origin knowledge would also require repeated derivation of breed-specific SNP effects.

In addition to genomic prediction analyses, knowledge of breed-of-origin of alleles can also be used in genome-wide association studies (GWAS), accounting for the fact that the effect of causative mutations on phenotypes may depend on breed-of-origin. The approach can be similar to that using parental origin of sequence variants [24], in which genomic imprinting restricts the effect to the allele inherited from a parent of a specific sex; however, to be able to distinguish between parental origin and breed-of-origin, reciprocal crosses will be needed.

The genomic prediction model and GWAS that account for breed-of-origin can also be tested using haplotypes instead of single SNPs, which can increase prediction accuracies in genomic prediction [25], and increase power and precision in GWAS [26]. However, although the output of the breed-of-origin approach provided 18 haplotypes libraries, it will still be necessary to combine them and redefine the start and endpoints of the haplotypes so that they are suitable for these types of analyses.

## Conclusions

Breed-of-origin of alleles of crossbred animals can be empirically derived without pedigree information. Pedigree information is, however, useful to reduce computation time and slightly improves assignment percentage. Around 94 % of the alleles of three-breed cross pigs were assigned a breed-of-origin. The results of this approach for assigning breed-of-origin to alleles allows the use of models that implement breed-specific effects of SNP alleles in genomic prediction, with the aim to improve selection of purebred animals for crossbred offspring performance. Breed-of-origin information also opens new possibilities to study associations between SNPs and production traits.

## Additional file

10.1186/s12711-016-0234-9 Allele assignment (%) per chromosome (Chr) to synthetic boar (S), Landrace (LR), or Large White (LW) as breed-of-origin when using pedigree information and a relaxation factor of 20 %. The data provided represent the percentage of allele assignment to each purebred line as breed-of-origin per chromosome when using pedigree information and a relaxation factor of 20 %.
