# Supplementary material for: Empirical determination of breed-of-origin of alleles in three-breed cross pigs
Source: Genet Sel Evol. 2016 Aug 4;48:55. doi: 10.1186/s12711-016-0234-9 (PMC4973529; doi:10.1186/s12711-016-0234-9)
Supplement: Supplementary file 1 — 10.1186/s12711-016-0234-9 Allele assignment (%) per chromosome (Chr) to synthetic boar (S), Landrace (LR), or Large White (LW) as breed-of-origin when using pedigree information and a relaxation factor of 20 %. The data provided represent the percentage of allele assignment to each purebred line as breed-of-origin per chromosome when using pedigree information and a relaxation factor of 20 %. [file 12711_2016_234_MOESM1_ESM.docx]

## Additional file 1. Allele assignment (%) per chromosome (Chr) to synthetic boar (S), Landrace (LR), or Large White (LW) when using pedigree information and a relaxation factor of 20%.

| **Chr** | **Paternal** | **Maternal** | | | **Total** |
| --- | --- | --- | --- | --- | --- |
|  | **Line S** | **Line LR** | **Line LW** | **Total** |  |
| 1 | 48.52 | 22.83 | 22.24 | 45.07 | 93.59 |
| 2 | 49.78 | 21.68 | 22.43 | 44.11 | 93.89 |
| 3 | 49.84 | 23.35 | 22.42 | 45.76 | 95.60 |
| 4 | 49.81 | 23.31 | 22.40 | 45.71 | 95.52 |
| 5 | 49.26 | 23.85 | 22.61 | 46.46 | 95.72 |
| 6 | 49.66 | 22.78 | 23.54 | 46.32 | 95.98 |
| 7 | 49.49 | 23.31 | 22.40 | 45.71 | 95.20 |
| 8 | 49.78 | 22.94 | 23.04 | 46.00 | 95.78 |
| 9 | 49.80 | 23.46 | 22.27 | 45.73 | 95.53 |
| 10 | 49.84 | 22.66 | 22.55 | 45.21 | 95.05 |
| 11 | 49.66 | 24.28 | 21.86 | 46.14 | 95.80 |
| 12 | 49.26 | 22.60 | 21.71 | 44.32 | 93.58 |
| 13 | 48.52 | 22.83 | 22.24 | 45.07 | 93.59 |
| 14 | 49.70 | 22.10 | 23.02 | 45.10 | 94.80 |
| 15 | 49.77 | 22.91 | 23.35 | 46.26 | 96.03 |
| 16 | 49.81 | 22.03 | 23.93 | 45.97 | 95.77 |
| 17 | 49.77 | 23.58 | 23.09 | 46.68 | 96.45 |
| 18 | 49.77 | 23.25 | 23.22 | 46.47 | 96.24 |
| Total | 49.56 | 22.99 | 22.68 | 45.67 | 95.23 |
